# Supplementary material for: MYCN-induced nucleolar stress drives an early senescence-like transcriptional program in hTERT-immortalized RPE cells
Source: Sci Rep. 2021 Jul 14;11:14454. doi: 10.1038/s41598-021-93863-9 (PMC8280219; doi:10.1038/s41598-021-93863-9)
Supplement: Supplementary file 1 — Supplementary Information 1. [file 41598_2021_93863_MOESM1_ESM.pdf]

Supplemental information for "MYCN-induced nucleolar stress drives an early senescence-like transcriptional program in hTERT-immortalized RPE cells"

Sofia Zanotti, Suzanne Vanhauwaert, Christophe Van Neste, Volodimir Olexiouk, Jolien Van Laere, Marlies Verschuuren, Joni Van der Meulen, Liselot M. Mus, Kaat Durinck, Laurentijn Tilleman, Dieter Deforce, Filip Van Nieuwerburgh, Michael D. Hogarty, Bieke Decaesteker, Winnok H. De Vos, Frank Speleman

Supplementary Fig. 1

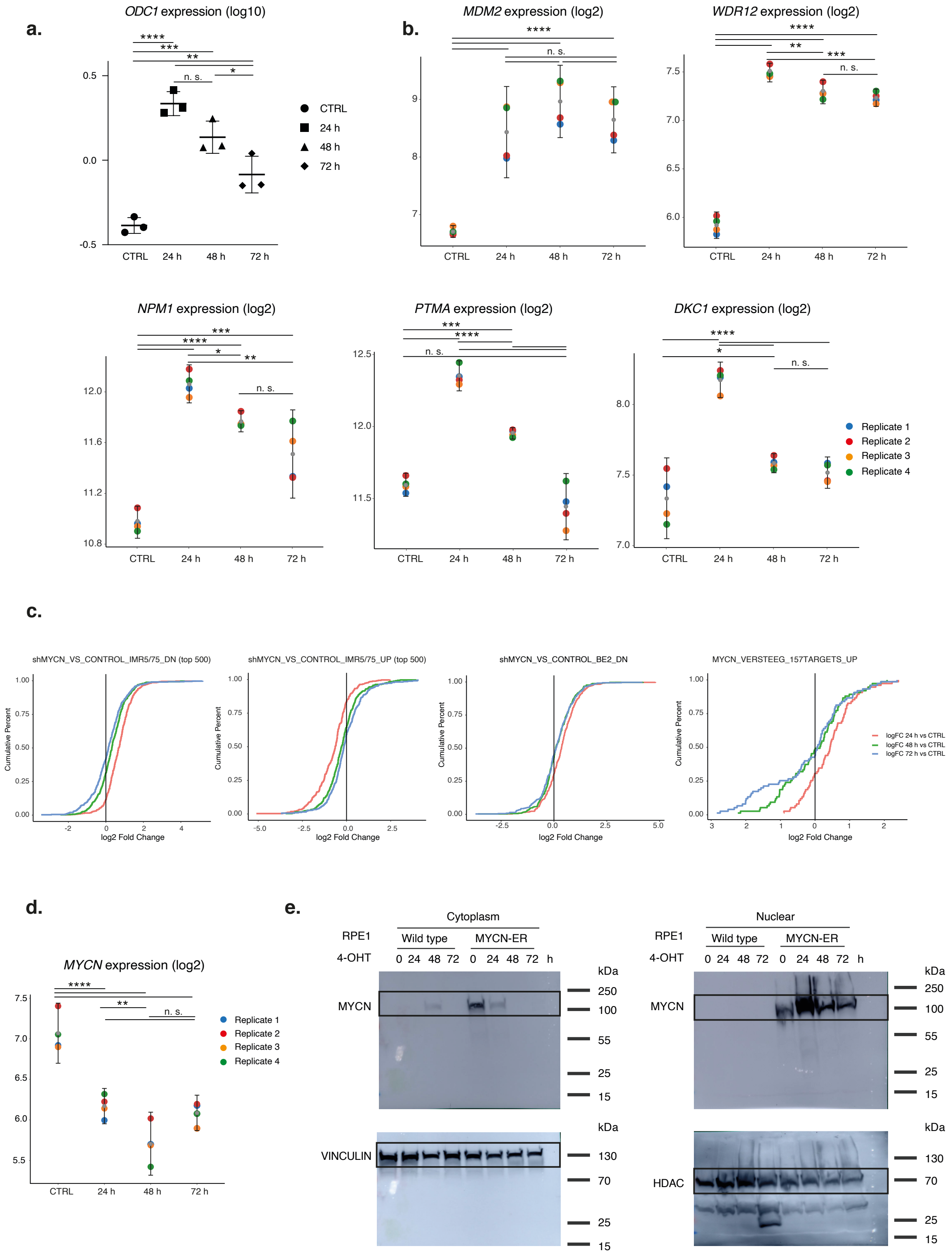

Supplementary Fig. 1. (**a**) log10 *ODC1* mRNA levels 24 h, 48 h, 72 h p.i.. Results are normalised to *18s*, *HMBS* and *SDHA* expression. Statistical differences examined by ANOVA

statistical analysis followed by a post-hoc Tukey's test for multiple comparisons for 24 h, 48 h, 72 h. Error bars represent the SD of the three biological replicates. (**b**) Log2 *MDM2*, *WDR12*, *NPM1*, *PTMA*, *DKC1* mRNA levels at 24 h, 48 h, 72 h p.i.. ANOVA statistical analysis followed by a post-hoc Tukey's test for multiple comparisons. Error bars represent 95% CI of the four biological replicates (**c**) Cumulative distribution plot of the log fold changes of the public available gene signatures with shMYCN up- and downregulated genes in IMR-5/75 cells, shMYCN downregulated genes in SK-N-BE(2c) cells and MYCN targets in IMR-32 cells for the 24 h vs CTRL, 48 h vs CTRL and 72 h vs CTRL comparisons in RPE-1-MYCN-ER cells. (**d**) log2 *MYCN* mRNA levels in MYCN-ON cells (**e**) Uncropped blots of Fig. 1a-b. Error bars represent the 95% CI of the four biological replicates. (n.s.= not significant, * = p-value < 0, 05; ** = p-value < 0, 01; *** = p-value < 0, 001).

Supplementary Fig. 2

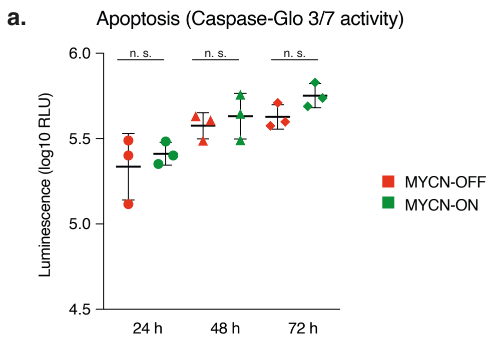

Supplementary Fig. 2. (**a**) Caspase-Glo 3/7 luminescent assay on MYCN-ON and OFF cells per time points indicated. Error bars represent the SD of the three biological replicates. (n.s.= not significant).

Supplementary Fig. 3

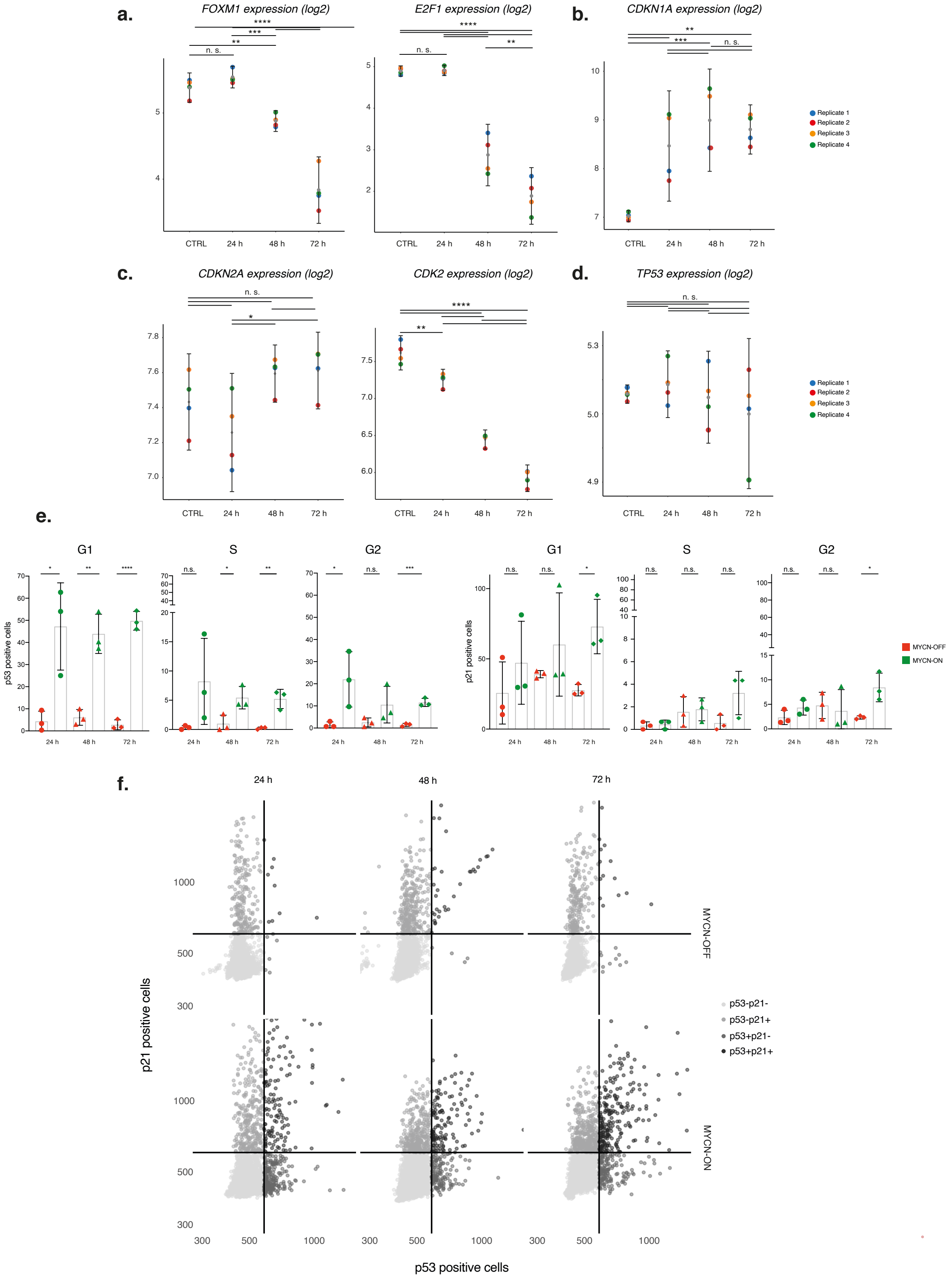

Supplementary Fig. 3. log2 *FOXM1*, *E2F1* (**a**), *CDKN1A* (**b**), *CDKN2A*, *CDK2* (**c**) and *TP53* (**d**) mRNA levels in MYCN-ON cells. ANOVA statistical analysis followed by a post-hoc

Tukey's test for multiple comparisons. Error bars represent the 95% CI of the four biological replicates (n.s.= not significant, * = p-value < 0, 05; ** = p-value < 0, 01; *** = p-value < 0, 001). (**e**) Individual quantifications of p21 (left) and p53 (right) per cell cycle phase shows that p21 is overexpressed in G1 whereas p53 is up in all phases in MYCN-ON cells. (**f**) Scatterplot of p21 and p53 protein expression quantification shows accumulation for both proteins in MYCN-ON cells for all time points. (n.s.= not significant, * = p-value < 0, 05; ** = p-value < 0, 01; *** = p-value < 0, 001).

Supplementary Fig. 4

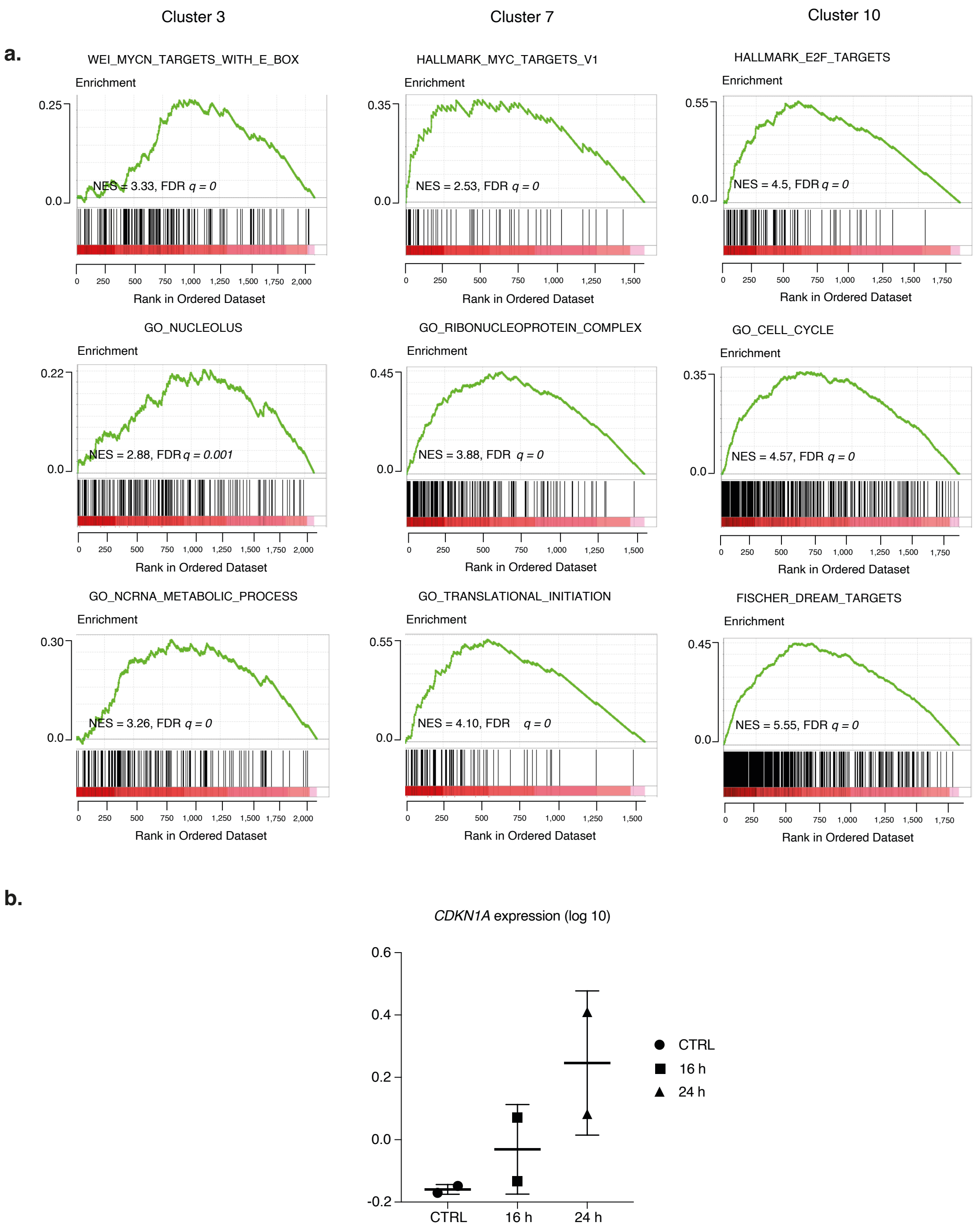

Supplementary Fig. 4. (**a**) Gene Set Enrichment analysis on cluster 3,7 and 10 revealed enrichment for MYCN signatures and gene sets associated with ribosome biogenesis in cluster 3 and 7, while cluster 10 is enriched for FOXM1, DREAM, and E2F targets related to cell cycle. (**b**) log10 *CDKN1A* mRNA levels 16 h and 24 h p.i.. Results are normalised to *18s*, *HMBS* and *SDHA* expression. Error bars represent the SD of the two biological replicates.

Supplementary Fig. 5

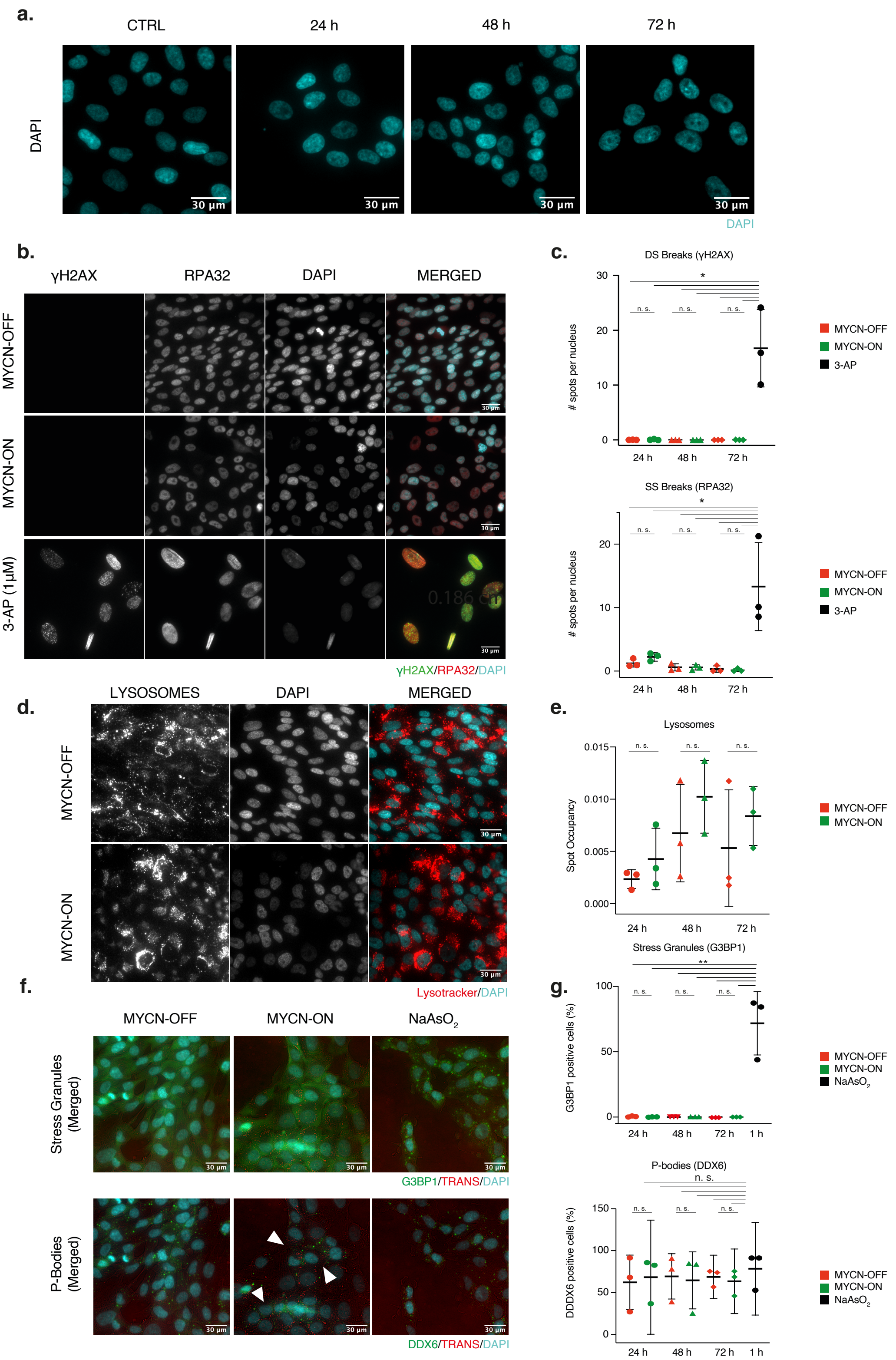

Supplementary Fig. 5. (**a**) Representative images of DAPI channel indicating no induction of DAPI-dense nuclear foci. (**b**) Immunofluorescence for γH2AX (green) and RPA32 (red) foci

show MYCN-ON cells do not accumulate DNA damage. (**c**) Quantification of γH2AX (top) and RPA32 (bottom), expressed by the number of foci per nucleus. (**d-e**) Imaging analysis of lysosomes show no significant increase between MYCN-ON and MYCN-OFF cells. (**f**) immunofluorescence staining for G3BP1 (marker for Stress Granules) (top; green) and DDX6 (marker for P-bodies) (bottom; green) merged with inverted brightfield (red) and DAPI (cyan). (**g**) Imaging analysis of stress granules (top) and P-bodies (bottom). Error bars represent the SD of the three biological replicates. Statistical differences examined by unpaired Student's t-test. (n.s.= not significant, * = p-value < 0, 05; ** = p-value < 0, 01; *** = p-value < 0, 001). Scale bar = 30 µm. Images were visualised and annotated in Fiji (v.2.0.0-rc-69/1.52p; http://imagej.net/).
